# Psychometric validation of the Arabic WHO Ageism Experiences Scale in Libya: evidence from a humanitarian context

**DOI:** 10.1093/ageing/afag083

**Published:** 2026-05-04

**Authors:** Xuefei Li, Ahmed Alameen, Raja Alagaili, Jebril Gebril, Maya Abi Chahine, Aidan Timlin, Aja Louise Murray

**Affiliations:** Department of Psychology, University of Edinburgh, Edinburgh, UK; Alsafwa Charity Organization, Tripoli, Libya; University of Tripoli, Tripoli, Tripoli District, Libya; Alsafwa Charity Organization, Tripoli, Libya; University of Tripoli, Tripoli, Tripoli District, Libya; Alsafwa Charity Organization, Tripoli, Libya; National Center for Disease Control, Tripoli, Tripoli District, Libya; HelpAge International, London, UK; University for Seniors Program, American University of Beirut, Beirut, Beirut Governorate, Lebanon; HelpAge International, London, UK; Department of Psychology, University of Edinburgh, Edinburgh, UK

**Keywords:** ageism, WHO Ageism Experiences Scale, psychometric validation, older people, disaster

## Abstract

Ageism against older adults is associated with a range of negative consequences, including poorer health and well-being outcomes. However, systematic reviews have identified that there is a lack of well-validated measures that capture ageism according to contemporary ageism definitions and that can be used to illuminate ageism experiences across the world. To address this gap, the WHO Ageism Experiences Scale was developed; however, to date it has not been validated in Arabic and there is limited knowledge about its functioning in humanitarian settings, where issues related to ageism may be exacerbated. The present study, therefore, conducted the first psychometric evaluation of the new Arabic translation of the scale in Libya, including participants who were directly affected by the recent devastating floods. As relevant, results supported the factorial validity, internal consistency reliability, convergent validity and concurrent validity of the subscale scores, including in participants affected by the floods. This suggests that the Arabic version of the scale can be recommended for use by researchers to advance the evidence base on how ageism and its consequences may be exacerbated in humanitarian settings, and by humanitarian organisations and policymakers to inform postdisaster needs assessments and programme evaluations in Arabic-speaking and humanitarian contexts.

## Key Points

- This is the first psychometric validation of the WHO Ageism Experiences Scale in Arabic, one of the six official UN languages.
- The study provides the first validation of the scale in a humanitarian disaster setting, among older adults in Libya affected by the 2023 floods.
- The findings support the validity, internal consistency reliability, convergent validity and concurrent validity of the scale.
- Ageism scores correlated with PTSD symptoms and secondary stressors among flood-affected participants.
- This study facilitates ageism research across diverse Arabic-speaking contexts.

## Introduction

Ageism is a multidimensional concept comprising stereotypes, prejudice and discrimination towards people due to their age, with manifestations at individual (self-directed), interpersonal and institutional levels [1]. Ageism against older adults is associated with a wide range of adverse outcomes, including poorer health outcomes [2, 3] and substantial economic costs [4]. As part of a World Health Organization (WHO)-led campaign, a comprehensive review highlighted that a key barrier to progress on tackling ageism was a lack of instruments that can be used to measure it in accordance with contemporary definitions and which have validity across and within diverse country settings [5]. Several systematic reviews since this seminal review, have further reinforced this conclusion [3, 6, 7].

To address this need, the WHO Ageism Experiences Scale [8] was developed through a rigorous multistage process. The scale has been validated in the contexts of a growing number of countries [9, 10]; however, to date, it has not been validated in Arabic or in a country in the Eastern Mediterranean Region (EMRO): one of the six major world regions defined by WHO, spanning the Middle East, North Africa and Western Asia. The region has been described as one of the most ‘tumultuous’ of the WHO regions and has faced significant challenges related to conflict, climate and associated health impacts [11].

Libya, one of the 21 member states of the EMRO region has experienced a prolonged period of political conflict and in 2023 was hit by devastating floods that resulted in thousands of deaths and the destruction of homes and critical infrastructure. Ageism, like other forms of hostility towards more marginalised groups, can become exacerbated when strains are placed on societies [12] and is thought to be a factor in older adults being disproportionately affected by disasters. Self-directed ageism can also affect the decisions of older adults to deprioritise their needs relative to those of younger generations [12, 13]. This makes it essential that a measure of ageism is available that can be used in humanitarian settings.

The current study provides the first validation of the WHO Ageism Experiences Scale in the context of a natural disaster, among people living in Libya who were directly affected by the floods. A second important contribution is to provide the first validation of the Arabic version of the scale, one of the six official UN languages, with 422 million speakers (native and non-native). There are also 25 Arabic-speaking countries that claim Arabic as an official or co-official language [14].

## Methods

### Participants

Participants (*n* = 350) were older adults (over 60 years old) from Libya, divided into two groups: directly affected by the floods in Derna city (*n* = 175), labelled ‘affected’, and not directly affected, labelled ‘unaffected’ (*n* = 175). Participants were required to be aged 60 years or above; individuals unable to provide informed consent or complete the interview due to severe cognitive or physical impairment were excluded. To ensure broad geographic representation while maintaining feasibility in a postdisaster environment, the overall sampling approach was quota-based convenience sampling, a form of nonprobability sampling. The process began with establishing quotas based on the municipal neighbourhood classification of Derna. The city was divided into its officially recognised areas, and participants were selected proportionally from each area to reflect the overall distribution of older adults across the city. However, due to the humanitarian context, specifically the devastation caused by the floods and the lack of a comprehensive sampling frame, strict random selection was not possible. Therefore, recruitment within each quota relied on convenience sampling through local community networks. Data collection was carried out through interviews administered by trained field staff. Although the sampling frame did not allow for *a priori* stratification by individual demographic factors beyond geography, the recruitment strategy—relying on established community networks within the proportional quotas—resulted in a sample with notable diversity. This was evident across key demographic variables, including sex, age, educational attainment and subjective social status. This spontaneous diversity ensured that the sample was sufficiently varied to examine the influence of these factors on ageism experiences.

There were 124 males and 51 females in the affected group, with a mean age of 67.57 (SD = 5.52, range = 60–87). Participants in this group varied in educational level: 7 held a master’s degree (or equivalent), 54 held a bachelor’s degree (or equivalent), 51 had completed tertiary school, 39 had completed secondary school, 9 had completed primary school, 14 were illiterate and 1 reported an educational level classified as ‘other’.

There were 128 males and 47 females in the unaffected group, with a mean age of 68.73 (SD = 6.20, range = 60–89). Educational levels in this group were as follows: 61 held a bachelor’s degree or equivalent, 48 had completed tertiary school, 31 had completed secondary school, 12 had completed primary school and 17 were classified as illiterate.

### Measures

Full details of the measures are provided in the Supplementary Materials, and the items comprising our core measure, the WHO Ageism Experiences Scale, are presented in Table 1. In brief, the WHO Ageism Experiences Scale [8], Perceived Ageism Questionnaire (PAQ) [15], SF-12v2 [16], General Health Questionnaire, 12-item version (GHQ-12) [17]*,* WHO-5 Wellbeing Index [18], UCLA Loneliness Scale [19]*,* contact quantity and quality with other age groups [20], a four-item abbreviated version of the Posttraumatic Stress Disorder (PTSD) Checklist for DSM-5 [21], Patient Health Questionnaire [22], Generalised Anxiety Disorder 2-item scale [23, 24] and a measure of flood-related stressors based on 18 items (provided in Supplementary Table S1), originally utilised in a prior study [25], were administered. The WHO Ageism Experiences Scale was the focal measure, and all others were included as convergent and concurrent validity measures. Participants reported their age (in years), gender (male, female, not listed, please specify), level of education and perceived social status. To ensure contextual appropriateness, the education level response options were developed in collaboration with local researchers.

**Table 1:** Item contents for the WHO Ageism Experiences Scale.

Item number	Content domain	Keying	Item
**1**	Self-stereotypes	−	At my age, my life has plenty of purpose
**2**	Self-stereotypes	+	I am a burden because of my age
**3**	Self-prejudice	*−*	I am embarrassed of my age
**4**	Self-discrimination	−	Due to my age, I limit my participation in discussions even when they are about things that affect me
**5**	Self-discrimination	−	There are things I would like to do if I did not consider them inappropriate for my age group
**6**	Interpersonal stereotypes	−	Others think that I have nothing valuable to contribute to society because of my age
**7**	Interpersonal stereotypes	+	Others think that at my age I am able to make decisions for myself
**8**	Interpersonal prejudices	−	Others feel frustrated with me due to my age
**9**	Interpersonal prejudices	−	Other people feel uncomfortable around me because of my age
**10**	Interpersonal discrimination	−	Due to my age, other people talk to me as if I need things simplified
**11**	Interpersonal discrimination	−	Others make decisions for me because of my age
**12**	Interpersonal discrimination	−	Due to my age, others make me feel excluded
**13**	Institutional discrimination	−	Policies made by the government (e.g. on housing, social security, healthcare) do not meet the needs of people my age
**14**	Institutional discrimination	+	People my age are portrayed positively in the media
**15**	Institutional discrimination	−	I have been turned down for an opportunity (e.g., a job or volunteering opportunity) that I was qualified for because of my age

*Note.* ‘Keying’ refers to the positive versus negative wording of the items. Positively keyed items were reverse-scored before forming composite scores, so higher composite scores reflect higher levels of experienced ageism.

### Ethics

This study received ethical approval from the University of Edinburgh’s School of Philosophy, Psychology and Language Sciences (PPLS) Research Ethics Committee (ref: 241-2425/2) and from the relevant local ethics committees at the data collection sites. Prior to participation, informed consent was obtained from all the participants.

### Statistical procedure

#### Overview

Given our goal of evaluating the psychometric properties of the WHO Ageism Experiences scale (i) in Arabic and (ii) in the context of humanitarian emergencies, all the analyses were conducted both overall and stratified by group: directly affected by the floods versus not directly affected by the floods.

The WHO Ageism Experiences Scale consists of three domains: self-directed, interpersonal and institutional ageism. For the self-directed ageism subdomain, reflective indicators are assumed, i.e. that an underlying latent psychological variable is assumed to ‘cause’ the individual items responses [26]. For these, it is appropriate to assess factor structure. For the domains of interpersonal and institutional ageism, formative indicators are assumed, meaning no underlying latent variable is posited. Instead, it is assumed that people may be exposed to ageism from varying sources with heterogeneous causes rather than a single underlying latent cause. As such, latent-variable methods are not applicable for these sets of items. For these items, only analyses examining sociodemographic patterning of scores and associations with other variables are conducted.

#### Confirmatory factor analysis

Previous research has suggested a single-factor model for the self-directed ageism items; therefore, we fitted a confirmatory factor analysis (CFA) model with items associated with a single latent factor. The model was estimated with maximum likelihood estimation in the lavaan R package [27]. Scaling and identification were achieved by fixing the latent variable variance to 1. Good fit was defined based on comparative fit index (CFI) and Tucker–Lewis index (TLI) values > 0.95 and root mean square error of approximation (RMSEA) and standardised root mean square residual (SRMR) values < 0.08. If the models did not fit well, we examined modification indices (MIs) and expected parameter changes (EPCs) to identify potential sources of mis-fit.

#### Internal consistency reliability

McDonald’s omega total [28] was used to assess the internal consistency reliability of the self-directed ageism subscale. In addition, the standard error of measurement (SEM) [29] was calculated to quantify measurement error in the observed scores. While reliability coefficients reflect the proportion of variance attributable to true scores, they are less informative at the individual level [30]. The SEM expresses measurement error in the same unit as the original scale, providing an index of the precision of individual observed scores and allowing confidence intervals to be derived around individual scores where required [31].

#### Sociodemographic patterning

We examined the sociodemographic patterning of the WHO Ageism Experiences Scale scores by sex, age and educational level. Univariate linear regressions with the WHO Ageism Experiences overall and self-directed, interpersonal and institutional scores as the outcome and each sociodemographic variable as the predictor were fitted.

#### Convergent validity

We examined the Pearson correlations between the WHO Ageism Experiences Scale scores and the PAQ subscale scores. Correlation coefficients after correction for attenuation were also obtained for self-directed ageism. We anticipated that the negative ageism scores would be more highly correlated with the WHO Ageism Scale scores than the positive ageism scores given that the latter focuses on negative ageism. Further, given that the PAQ items focus on interpersonal ageism, we anticipated that the interpersonal ageism scores from the WHO Ageism Scale would be more highly correlated with the PAQ scores than the self-directed and institutional ageism scores.

#### Concurrent validity

We examined the Pearson correlations between the WHO Ageism Experiences Scale scores and a range of outcomes that have previously established relations with ageism. This includes mental health outcomes: anxiety, depression, general psychological distress and well-being, loneliness, intergenerational contact and physical health [1].

To explore the relevance of the WHO Ageism Experiences Scale in the current humanitarian context, we additionally examined the correlations with PTSD symptom scores and secondary stressors. We hypothesised that ageism may be related to stronger impacts of humanitarian disasters and to therefore be correlated with higher PTSD and secondary stressors scores.

## Results

### Descriptive statistics

Item-level descriptive statistics for each item overall and by group are provided in Table 2. These suggested that for all the items, respondents in both groups utilised the full range of the response options. The highest levels of ageism were reported in relation to institutional discrimination, i.e. ‘Policies made by the government (e.g., on housing, social security, healthcare) do not meet the needs of people my age,’ self-directed discrimination, i.e. ‘Due to my age, I limit my participation in discussions even when they are about things that affect me’ and ‘There are things I would like to do if I did not consider them inappropriate for my age group,’ interpersonal discrimination, i.e. ‘Due to my age, other people talk to me as if I need things simplified.’ This pattern suggests that experiences of discrimination, compared to stereotypes and prejudices, were most salient for this sample.

**Table 2:** Descriptive statistics for WHO Ageism Experiences Scale items.

Item	Overall					Affected					Unaffected				
	N	Mean	SD	Min	Max	N	Mean	SD	Min	Max		Mean	SD	Min	Max
**1**	331	2.81	1.12	1	5	165	2.65	1.05	1	5	166	2.96	1.17	1	5
**2**	337	2.27	0.98	1	5	166	2.22	0.97	1	4	171	2.32	0.99	1	5
**3**	342	1.94	0.95	1	5	174	1.87	0.94	1	5	168	2.01	0.96	1	5
**4**	343	3.37	1.17	1	5	171	3.28	1.19	1	5	172	3.47	1.14	1	5
**5**	335	3.20	1.04	1	5	165	3.16	0.99	1	5	170	3.24	1.09	1	5
**6**	331	2.78	1.11	1	5	166	2.71	1.16	1	5	165	2.84	1.07	1	5
**7**	317	2.87	1.17	1	5	159	2.90	1.19	1	5	158	2.84	1.15	1	5
**8**	306	2.37	0.97	1	5	155	2.39	0.94	1	5	151	2.35	1.01	1	5
**9**	315	2.46	0.91	1	5	157	2.44	0.86	1	5	158	2.47	0.95	1	5
**10**	328	3.14	0.99	1	5	162	3.00	1.03	1	5	166	3.27	0.94	1	5
**11**	333	2.23	1.09	1	5	166	2.13	1.06	1	5	167	2.32	1.11	1	5
**12**	322	2.42	0.99	1	5	165	2.33	1.03	1	5	157	2.52	0.94	1	5
**13**	291	3.98	1.09	1	5	151	3.93	1.09	1	5	140	4.04	1.09	1	5
**14**	303	2.65	0.90	1	5	156	2.67	0.90	1	5	147	2.63	0.90	1	5
**15**	305	2.98	0.97	1	5	151	2.95	0.95	1	5	154	3.01	0.99	1	5

*Note.* Items scored as 1 = lowest levels of ageism to 5 = highest levels of ageism.

### CFA of self-directed ageism subscale

A one-factor CFA for the self-directed ageism items yielded fit statistics of CFI = 0.89, TLI = 0.79, RMSEA = 0.13 and SRMR = 0.06, overall falling short of the conventional criteria for good fit. The fit was also below conventional thresholds in both the affected (CFI = 0.93, TLI = 0.87, RMSEA = 0.10, SRMR = 0.05) and unaffected groups (CFI = 0.88, TLI = 0.75, RMSEA = 0.14, SRMR = 0.07), although the poor fit appeared particularly to relate to the latter group.

Inspection of MIs and EPCs in the model fit to the overall sample and group subsamples pointed to the inclusion of a residual covariance between items 2 and 3 (measuring perceiving oneself to be a burden and feeling embarrassed due to their age, respectively). Given that feeling a burden may engender feelings of shame or embarrassment, we judged it substantively meaningful to include this covariance. Doing so improved the fit of the model in the overall (CFI = 0.95, TLI = 0.86, RMSEA = 0.10, SRMR = 0.04), affected (CFI = 0.96, TLI = 0.90, RMSEA = 0.08, SRMR = 0.04) and unaffected (CFI = 0.97, TLI = 0.91, RMSEA = 0.08, SRMR = 0.05) samples. This revised model was judged acceptable given that it fitted well in the two subsamples. The poorer fit in the combined sample may indicate heterogeneity across the affected and unaffected samples, providing a counterindication to combining them in the same analysis. Standardised parameter estimates for this model are provided in Supplementary Table S2. All the loadings were salient (<|.30|) and statistically significant at *P* < .001.

#### Internal consistency reliability for the self-directed ageism scale

McDonald’s omega for the self-directed ageism scale was 0.69 for the overall sample, 0.69 for the affected group and 0.71 for the unaffected group. The SEM was 0.39 for the overall sample, 0.37 for the affected group and 0.38 for the unaffected group, suggesting comparable measurement precision across groups.

#### Sociodemographic patterning of ageism scores

Coefficients from the univariate regressions of the WHO Ageism Experiences Scale scores on sex, age and educational level are provided in Table 3. These suggested that in the overall sample, females experienced more interpersonal ageism than males and older age predicted self-directed, interpersonal and institutional experiences. Compared to having a bachelor’s degree–level education, being in the illiterate classification also predicted experiencing higher levels of all types of ageism. These patterns were mostly replicated in the affected and unaffected groups. The sex difference in ageism was exacerbated in the affected group for self-directed and interpersonal ageism.

**Table 3:** Sociodemographic patterning of WHO Ageism Scale scores.

Sociodemographic predictor	Self-directed		Interpersonal		Institutional	
	*B*	*P*	*B*	*P*	*B*	*P*
	**Overall sample**					
**Sex (reference = female)**	−0.03	.71	−0.17	.03^*^	−0.07	.42
**Age**	0.03	<.001^*^	0.03	<.001^*^	0.02	<.001^*^
**Educational level (reference = bachelor’s):**						
Illiterate	0.65	<.001^*^	0.57	<.001^*^	0.51	<.001^*^
Primary	0.04	.78	0.14	.36	0.56	<.001^*^
Secondary	0.06	.59	0.15	.13	0.25	.01^*^
Tertiary	0.10	.29	−0.01	.90	0.07	.46
Master’s	−0.15	.43	−0.03	.86	−0.23	.24
Other	0.78	.25	1.45	.02^*^	−0.06	.94
	**Affected group**					
**Sex (reference = female)**	−0.09	<.001^*^	−0.24	<.001^*^	−0.02	.84
**Age**	0.04	<.001	0.02	.02^*^	0.03	.004^*^
**Educational level (reference = bachelor’s):**						
Illiterate	0.84	<.001^*^	0.84	<.001^*^	0.42	.04^*^
Primary	0.04	.88	0.24	.33	0.68	.008^*^
Secondary	0.07	.63	0.15	.30	0.24	.10
Tertiary	0.11	.63	−0.02	.90	0.12	.35
Master’s	−0.02	.94	−0.28	.30	0.11	.68
	**Unaffected group**					
**Sex (reference = female)**	0.03	.78	−0.10	.32	−0.10	.37
**Age**	0.03	.002^*^	0.03	<.001^*^	0.02	.045^*^
**Educational level (reference = bachelor’s):**						
Illiterate	0.48	.01^*^	0.34	.04^*^	0.59	.003^*^
Primary	0.03	.88	0.06	.78	0.48	.03^*^
Secondary	0.08	.59	0.17	.21	0.28	.068
Tertiary	0.10	.45	0.00	.97	0.02	.89
Master’s	−0.28	.35	0.27	.30	−0.63	.04^*^

*Note.* Coefficients from each predictor are from separate univariate regression. ^*^Statistically significant at *P* < .05.

#### Convergent validity

Correlations between the self-directed, interpersonal and institutional ageism scores and the positive and negative ageism scores from the PAQ are provided in Table 4. Consistent with expectation and in support of convergent validity, the correlations were highest between the WHO Ageism Experiences interpersonal ageism scores and the PAQ Negative Ageism scores (ranging between 0.43 for the unaffected group and 0.52 for the affected group). The WHO Ageism Experiences subscale scores more broadly exhibited a pattern of correlations that was consistent with expectation: correlations were consistently higher with the PAQ negative ageism scores, compared with the PAQ positive ageism scores. Nevertheless, the majority of correlations were statistically significant between the WHO Ageism and Experiences and PAQ subscale scores. Only the correlations between WHO Ageism Scale Institutional ageism and the PAQ positive and negative subscale scores were nonsignificant and exclusively in the unaffected group.

**Table 4:** Convergent validity correlations.

	Overall sample				Affected group				Unaffected group			
	Positive ageism (PAQ)		Negative ageism (PAQ)		Positive ageism (PAQ)		Negative ageism (PAQ)		Positive ageism (PAQ)		Negative ageism (PAQ)	
	*r*	*P*	*r*	*P*	*r*	*P*	*r*	*P*	*r*	*P*	*r*	*P*
^**a**^ **WHO Ageism Scale Self-directed**	0.24 (.40)	<.001^*^	0.37 (.51)	<.001^*^	0.31 (.52)	<.001^*^	0.44 (.63)	<.001^*^	0.18 (.28)	.02^*^	0.29 (.39)	<.001^*^
**WHO Ageism Scale Interpersonal**	0.30	<.001^*^	0.47	<.001^*^	0.32	<.001^*^	0.52	<.001^*^	0.28	<.001^*^	0.43	<.001^*^
**WHO Ageism Scale Institutional**	0.16	<.001^*^	0.21	<.001^*^	0.19	<.001^*^	0.35	<.001^*^	0.14	.07	0.12	.12

^a^For self-directed ageism, values in parentheses represent correlation coefficients after correction for attenuation. These were calculated using the formula ${r}_{xy_{\mathrm{corrected}}}=\frac{r_{xy}}{\sqrt{\omega_x\ast{\omega}_y}}$, where ${\omega}_x$ and ${\omega}_y$ represent the McDonald’s omega reliability indices for the respective scales.

^*^Statistically significant at *P* < .05.

#### Concurrent validity

Correlations between the WHO Ageism Scale scores and established and hypothesised correlates of ageism are provided in Table 5. These suggested a pattern characterised by significant associations between ageism and a range of health and well-being outcomes, with only a few exceptions (e.g. the voluntariness and pleasantness of intergenerational contact). The strongest correlations were with loneliness and well-being (ranging from *r* = |0.36| to *r* = |0.49|).

**Table 5:** Concurrent validity associations.

	Self-directed ageism		Interpersonal ageism		Institutional ageism	
	*r*	*P*	*r*	*P*	*r*	*P*
**Overall sample**						
**General Health Questionnaire**	−0.16	.003^*^	−0.21	<.001^*^	−0.04	.435
**WHO Wellbeing**	−0.36	<.001^*^	−0.43	<.001^*^	−0.10	.067
**Loneliness**	0.42	<.001^*^	0.47	<.001^*^	0.09	.082
**Intergenerational contact frequency**	−0.20	<.001^*^	−0.27	<.001^*^	−0.12	.025^*^
**Intergenerational contact voluntariness**	0.03	.558	−0.02	.694	−0.02	.654
**Intergenerational contact quality**	−0.16	.004^*^	−0.28	<.001^*^	−0.13	.017^*^
**Intergenerational contact pleasantness**	−0.09	.084	−0.04	.414	−0.27	<.001^*^
**Physical health**	−0.31	<.001^*^	−0.25	<.001^*^	−0.22	<.001^*^
**Psychological health**	−0.40	<.001^*^	−0.38	<.001^*^	−0.20	<.001^*^
**PTSD**	0.23	<.001^*^	0.25	<.001^*^	0.28	<.001^*^
**General anxiety**	0.23	<.001^*^	0.25	<.001^*^	0.11	.038
**Depression**	0.15	.006^*^	0.18	.001^*^	−0.05	.396
**Secondary stressors**	0.12	.040^*^	0.12	.042^*^	0.01	.894
**Affected group**						
**General Health Questionnaire**	−0.22	.003^*^	−0.23	.002^*^	−0.10	.185
**WHO Wellbeing**	−0.33	<.001^*^	−0.49	<.001^*^	−0.15	.055
**Loneliness**	0.41	<.001^*^	0.46	<.001^*^	0.12	.126
**Intergenerational contact frequency**	−0.22	.003^*^	−0.36	<.001^*^	−0.18	.020^*^
**Intergenerational contact voluntariness**	−0.02	.768	−0.06	.431	−0.04	.587
**Intergenerational contact quality**	−0.16	.038^*^	−0.33	<.001^*^	−0.22	.004^*^
**Intergenerational contact pleasantness**	−0.14	.060	−0.05	.477	−0.27	<.001^*^
**Physical health**	−0.37	<.001^*^	−0.28	<.001^*^	−0.31	.001^*^
**Psychological health**	−0.44	<.001^*^	−0.40	<.001^*^	−0.26	.001^*^
**PTSD**	0.28	<.001^*^	0.19	.010^*^	0.35	<.001^*^
**General anxiety**	0.25	.001^*^	0.24	.001^*^	0.26	.001^*^
**Depression**	0.30	.000^*^	0.21	.005^*^	0.08	.275
**Secondary stressors**	0.15	.048^*^	0.15	.053	0.16	.035^*^
**Unaffected group**						
**General Health Questionnaire**	−0.09	.003^*^	−0.17	.002^*^	0.01	.185
**WHO Wellbeing**	−0.38	<.001^*^	−0.37	<.001^*^	−0.06	.055
**Loneliness**	0.40	<.001^*^	0.49	<.001^*^	0.07	.126
**Intergenerational contact frequency**	−0.17	.003^*^	−0.16	<.001^*^	−0.07	.020^*^
**Intergenerational contact voluntariness**	0.06	.768	0.01	.431	−0.01	.587
**Intergenerational contact quality**	−0.15	.038^*^	−0.20	<.001^*^	−0.03	.004^*^
**Intergenerational contact pleasantness**	−0.04	.060	−0.03	.477	−0.27	<.001^*^
**Physical health**	−0.28	<.001^*^	−0.24	<.001^*^	−0.15	.001^*^
**Psychological health**	−0.34	<.001^*^	−0.35	<.001^*^	−0.14	.001^*^
**PTSD**	0.20	<.001^*^	0.33	.010^*^	0.20	<.001^*^
**General anxiety**	0.21	.001^*^	0.27	.001^*^	0.02	.001^*^
**Depression**	0.01	<.001^*^	0.14	.005^*^	−0.14	.275
**Secondary stressors**	0.20	.048^*^	0.08	.053	−0.08	.035^*^

*Note.* * = significant at *p* <.05.

In the flood-affected group, ageism was correlated with outcomes related to the impact of the floods. PTSD scores were significantly correlated at *r =* 0.28, *r* = 0.19 and *r* = 0.35 with self-directed, interpersonal and institutional ageism, respectively. Self-directed (*r =* 0.15) and institutional ageism (*r =* 0.16) were also significantly correlated with secondary flood-related stressors.

## Discussion

The current study provides the first psychometric validation of the new WHO Ageism Experiences Scale in Arabic (the fifth most spoken language globally) and in a humanitarian disaster context. Our psychometric analyses suggested that the scale scores had good properties in two subsamples of participants: those who were versus were not directly affected by the 2023 floods in Libya. Specifically, factorial validity, internal consistency reliability, convergent validity and concurrent validity were majority supported. Further, in the flood-affected group, PTSD scores and secondary stressors associated with the floods were correlated with WHO Ageism Experiences Scale scores, demonstrating their relevance in the context of a natural disaster.

The availability of a validated Arabic version is a significant step in the availability of a measure of ageism experiences that can be used and compared across the world. While assessments of cross-cultural applicability and linguistic translatability were integral to the development and content validity assessments of the scale [32], it is only through empirical testing in local populations that the validity of the scale scores in different languages and contexts can be verified. The process of translation and piloting and the empirical psychometric results suggested that the scale could be translated and implemented in Arabic with minimal issue. However, our empirical analyses highlighted some items where missingness rates were high, which may reflect ambiguities, lower cultural relevance or—in the case of the item regarding institutional ageism in policies—a reluctance to criticise the government. Future research using cognitive interviewing may be valuable for further illuminating these results.

The availability of the scale in Arabic may provide a starting point for increased ageism research in Arabic-speaking countries where there is currently limited availability of measures of ageism, reflecting the general bias towards measures developed and validated in English [5, 7]. Correspondingly, there has been relatively little research on ageism in Arabic-speaking participants, with a few exceptions; see e.g. Abdelkader *et al.* and Alhajj *et al.* [33, 34]. However, researchers should be cautious when generalising the findings or using the scale in nonhumanitarian settings, as this validation was specifically conducted in a disaster-affected region. Although this study also included validation among a nonaffected population, further evidence is still required from stable, nondisaster settings to confirm its optimal functioning across different general populations in Arabic-speaking countries. Further, given the vast cultural diversity of Arabic-speaking country settings, it will be important to conduct further validations across different populations.

The results also suggested that the scale performed reasonably in participants who were affected by the 2023 floods in Libya. A descriptive comparison suggests that the psychometric properties were comparable for those who were or were not directly affected by the floods. Further, the scores were correlated with variables related to the impact of the floods (PTSD scores and secondary stressors). Together, this suggests that the scale may be a useful tool for illuminating experiences of ageism in the context of humanitarian settings, facilitating the further development of the evidence base on the drivers, consequences and solutions to ageism in such contexts where ageism may increase and its consequences may be magnified [12, 13]. The issue of ageism in the context of natural disasters in particular is likely to become more pressing given population ageing trends combined with a projected increase in climate-change-related natural disasters [35].

### Limitations and future directions

It is important to note the limitations of the present study. First, while our sample was diverse in terms of sociodemographics and ageism experiences, the use of nonprobability sampling means it is not strictly population-representative. This methodology, though necessary in the postdisaster humanitarian setting given the lack of an up-to-date sampling frame, meant that selection was nonrandom within the geographical quotas, which may have introduced some selection bias (e.g. towards more accessible or connected individuals). Second, we were not able to assess all the psychometric properties that may be relevant for the scale [36], such as sensitivity to intervention effects and test–retest reliability. It will also be important to conduct further psychometric validations of the Arabic version of the WHO Ageism Scale in different populations, including different country settings. Future research could conduct measurement invariance analyses across different country settings and key subgroups such as sex and gender, age and ethnicity: analyses we were unable to perform due to the limited sample size of our subgroups. As ageism is relevant for all ages, it would also be valuable to conduct validations of the scale in younger age groups, as the scale was designed to capture ageism experiences from adolescence upwards.

## Conclusions

The WHO Ageism Experiences Scale Arabic version can be recommended for use. It performed reasonably in participants affected by a natural disaster and correlated with impacts of the natural disaster. This suggests that the scale may additionally be a valuable tool for illuminating ageism in the context of humanitarian settings, where ageism and its impacts may be exacerbated.

## Supplementary Material

Supplementary_materials_afag083
