# Supplementary material for: Psychometric validation of the Arabic WHO Ageism Experiences Scale in Libya: evidence from a humanitarian context
Source: Age Ageing. 2026 May 4;55(5):afag083. doi: 10.1093/ageing/afag083 (PMC13137322; doi:10.1093/ageing/afag083)
Supplement: Supplementary_materials_afag083 [file supplementary_materials_afag083.docx]

**Psychometric Validation of the Arabic WHO Ageism Experiences Scale in Libya: Evidence from a Humanitarian Context**

**Supplementary Materials**

**Full Details of Measures**

***WHO Ageism Experiences Scale***

The *WHO Ageism Experiences Scale* is a 15-item scale measuring ageism experiences in terms of self-directed, interpersonal and institutional ageism, covering the domains of stereotypes, prejudices, and discrimination. Here, stereotypes refer to cognitions, prejudices to affect and discrimination to behaviours directed towards people based on their age. The items were selected from the *WHO Ageism item pool*, developed to meet the need for a new psychometrically robust measure of ageism that was globally applicable, could measure ageism against different age groups, and which could capture all core dimensions of ageism as recognised in contemporary ageism theory [1]. The item contents are shown in Table 1 and include a mixture of positively and negatively worded items. Responses are provided in a 5-point scale from *strongly agree* to *strongly disagree* with a ‘don’t know or not applicable’ option also offered. Previous research has supported the psychometric properties of the scale in different languages and settings [2]

We adopted a translation developed by the Late Life Learning, Cognition and Aging (3LC) Study (<https://www.aub.edu.lb/3lc/Pages/default.aspx>). The team used an adapted version of the gold standard ‘Translation, Review, Adjudication, Pretesting, and Documentation’ (TRAPD) method [3]. The Arabic translation completed by 3LC Lebanon team was reviewed by a professional Libyan translator who suggested few minor edits in line with the Libya culture and Arabic dialect.

***Perceived Ageism Questionnaire***

The *Perceived Ageism Questionnaire* (PAQ) [4] is an additional measure of ageism, included in the present study to permit an assessment of convergent validity. As there is no pre-existing gold standard measure of ageism, its selection was based on its inclusion of items measuring both stereotypes and discrimination, i.e., its coverage of two of the three dimensions recognised in contemporary ageism theory. It is an 8-item measure with content covering both negative and positive ageism. It was originally developed in the Netherlands but has been translated into English. For the present study the English translation was used as the source for the translation into Arabic. In previous psychometric evaluations, a two-factor structure has been suggested, reflecting minimally correlated positive and negative ageism concepts. The internal consistency reliability values for the corresponding subscales have been found to be good (negative: Cronbach’s alpha = .75; positive: Cronbach’s alpha = .81).

***SF-12v2***

The SF-12v2 is a widely validated, abbreviated version of the SF-36 Health Survey that measures health-related quality of life. The instrument consists of 12 items that yield two summary scores: the Physical Component Summary (PCS) and Mental Component Summary (MCS) [5]. Items are scored on a five-point Likert-type scale from ‘all of the time’ to ‘none or the time’.

***General Health Questionnaire***

The General Health Questionnaire 12 item version (GHQ-12) is a brief screening instrument for detecting non-psychotic psychiatric disorders and psychological distress in community and clinical settings. It comprises 12 items referring to concentration, sleep, playing a useful role, feeling capable of making decisions, feeling constantly under strain, having problems overcoming difficulties, enjoying day-to-day activities, feeling able to face problems, feeling unhappy or depressed, losing confidence, feeling worthless, and general happiness. Responses to each item are on a four-point Likert-type scale from ‘better than usual’ to ‘much less than usual’, with higher scores suggest greater psychological distress.

***WHO Well-being Index***

*Wellbeing* was assessed using the WHO-5 Wellbeing Index [6], a widely employed measure of subjective psychological well-being. The index comprises five items capturing the domains of cheerfulness, calmness, activity, restfulness, and interest. Participants respond on a six-point scale ranging from ‘all of the time’ to ‘at no time’.

***UCLA Loneliness Scale***

*Loneliness* was measured using a short form of the *UCLA Loneliness Scale* [7]*,*  the ULS-6, which has been validated in older adult populations previously [8]. This ULS-6 is a 6-item scale, with responses recorded on a 4-point scale from 1=never to 4=always.

***Contacts with other age groups***

*Contact quantity and quality with other age groups* will be measured using an adapted version of items used by Drury et al. (2016). Participants were asked to report their frequency, voluntariness, quality, and pleasantness of their interactions with children, adolescents, younger adults, middle-aged adults, and older adults.

***PTSD***

PTSD symptoms were assessed using the four-item abbreviated version of the PTSD Checklist for DSM-5 [10], Items were rated on a Likert scale from 1 (“not at all”) to 5 (“extremely”), with higher scores reflecting greater symptom severity. To assess symptoms specifically in relation to the flood, the original timeframe was modified to reference the event, with the prompt: “how much you have been bothered by that problem since September 2023.”

***PHQ-2***

Depression was assessed using the two-item Patient Health Questionnaire [11]. Each item had four response options: “not at all”, “several days”, “more than half the days”, and “nearly every day”. Higher scores reflected greater severity of depressive symptoms.

***GAD***

The Generalised Anxiety Disorder 2-item scale (GAD-2) is a brief screening instrument developed to identify anxiety disorders, particularly generalised anxiety disorder (GAD). It has demonstrated strong psychometric properties [12,13] and is often administered in conjunction with the PHQ-2.

***Flood-related secondary stressors***

The assessment of flood-related secondary stressors was based on 18 items, originally utilised in a prior study [14]. The full list of items is provided in Supplementary Table S1.

***Sociodemographic information***

Participants reported their age (in years), gender (male, female, not listed, please specify), level of education, and perceived social status. To ensure contextual appropriateness, the education level response options were developed in collaboration with local researchers.

**Table S1. Secondary stressors list**

|  | Situations Older Adults May Have Faced |
| --- | --- |
| SSF1 | Additional financial burdens |
| SSF2 | Potential or actual loss of employment or income |
| SSF3 | Additional work pressure (e.g., change of workplace) |
| SSF4 | Dealing with insurance issues |
| SSF5 | Repairing my home |
| SSF6 | Living in a damaged home |
| SSF7 | Having to move home permanently or temporarily |
| SSF8 | Loss of items with sentimental value |
| SSF9 | Concerns about mine and my family’s health |
| SSF10 | Jobs around the home/daily chores became more difficult |
| SSF11 | Changes in your child/children’s wellbeing or education |
| SSF12 | Relationship problems (e.g., arguing with partner/friends/children) |
| SSF13 | Spending more time helping family, friends, or community members |
| SSF14 | Loss of social activities |
| SSF15 | Disagreements/arguments with neighbours |
| SSF16 | Being separated from family members I normally live with |
| SSF17 | Concerns about the value of my house |
| SSF18 | Difficult decisions concerning pets |

**Table S2: Standardised parameter estimates for the self-directed ageism scale CFA**

|  | **Overall sample** | | **Affected group** | | **Unaffected group** | |
| --- | --- | --- | --- | --- | --- | --- |
| **Item** | **Loading** | ***p*** | **Loading** | ***p*** | **Loading** | ***p*** |
| 1 | 0.66 | <.001 | 0.47 | <.001 | 0.86 | <.001 |
| 2 | 0.55 | <.001 | 0.65 | <.001 | 0.47 | <.001 |
| 3 | 0.44 | <.001 | 0.38 | <.001 | 0.48 | <.001 |
| 4 | 0.50 | <.001 | 0.62 | <.001 | 0.38 | <.001 |
| 5 | 0.53 | <.001 | 0.55 | <.001 | 0.46 | <.001 |

*Note.* The residual correlation between items 2 and 3 was *r*=.27 (*p*=.001), *r*= .24 (*p*=.04), and *r*= .31 (*p=*.003) in the overall, affected, and unaffected subsamples respectively.

References

1. Murray AL, De La Fuente-Nunez V. Development of the item pool for the ‘WHO-ageism scale’: conceptualisation, item generation and content validity assessment. *Age and Ageing* 2023;**52**(Supplement_4):iv149–57.

2. Murray AL, Li X, Booth T. *Preliminary Validation of the 15-Item WHO Experiences of Ageism Scales in a Mixed-Age UK Sample*. 2024. https://osf.io/preprints/psyarxiv/7jcqk (11 Mar. 2025, date last accessed).

3. Walde P, Völlm BA. The TRAPD approach as a method for questionnaire translation. *Front Psychiatry* 2023;**14**:1199989. https://doi.org/10.3389/fpsyt.2023.1199989.

4. Brinkhof LP, Wit S de, Murre JM *et al.* The Subjective Experience of Ageism: The Perceived Ageism Questionnaire (PAQ). *International Journal of Environmental Research and Public Health* 2022;**19**(14):8792.

5. Fleishman JA, Selim AJ, Kazis LE. Deriving SF-12v2 physical and mental health summary scores: a comparison of different scoring algorithms. *Quality of Life Research* 2010;**19**(2):231–41.

6. Topp CW, Østergaard SD, Søndergaard S *et al.* The WHO-5 Well-Being Index: a systematic review of the literature. *Psychotherapy and Psychosomatics* 2015;**84**(3):167–76.

7. Russell DW. UCLA Loneliness Scale (Version 3): Reliability, validity, and factor structure. *Journal of Personality Assessment* 1996;**66**(1):20–40.

8. Neto F. Psychometric analysis of the short-form UCLA Loneliness Scale (ULS-6) in older adults. *European Journal of Ageing* 2014;**11**(4):313–9.

9. Drury L, Hutchison P, Abrams D. Direct and extended intergenerational contact and young people’s attitudes towards older adults. *British Journal of Social Psychology* 2016;**55**(3):522–43.

10. Blevins CA, Weathers FW, Davis MT *et al.* The Posttraumatic Stress Disorder Checklist for DSM-5 (PCL-5): Development and Initial Psychometric Evaluation. *Journal of Traumatic Stress* 2015;**28**(6):489–98. https://doi.org/10.1002/jts.22059.

11. Löwe B, Kroenke K, Gräfe K. Detecting and monitoring depression with a two-item questionnaire (PHQ-2). *Journal of Psychosomatic Research* 2005;**58**(2):163–71. https://doi.org/10.1016/j.jpsychores.2004.09.006.

12. Hlynsson JI, Carlbring P. Diagnostic accuracy and clinical utility of the PHQ-2 and GAD-2: a comparison with long-format measures for depression and anxiety. *Front Psychol* 2024;**15**. https://doi.org/10.3389/fpsyg.2024.1259997.

13. Kroenke K, Spitzer RL, Williams JBW *et al.* An Ultra-Brief Screening Scale for Anxiety and Depression: The PHQ–4. *Psychosomatics* 2009;**50**(6):613–21. https://doi.org/10.1016/S0033-3182(09)70864-3.

14. Tempest EL, English National Study on Flooding and Health Study Group, Carter B *et al.* Secondary stressors are associated with probable psychological morbidity after flooding: a cross-sectional analysis. *European Journal of Public Health* 2017;**27**(6):1042–7. https://doi.org/10.1093/eurpub/ckx182.
